# Effect of interleukin 21 and its receptor on CD8^+^ T cells in the pathogenesis of diffuse large B-cell lymphoma

**DOI:** 10.3892/ol.2014.2062

**Published:** 2014-04-11

**Authors:** ZHANSHAN CHA, HAIHUI GU, HUIJUN GUO, XIAOHUA TU, YAN ZANG, CHUNYAN ZHAO, MEIXIAN HUA, JAMES R. RECHLIC, LINDSAY M. OLASNOVA, HAIHAN SONG, BAOHUA QIAN

**Affiliations:** 1 Department of Transfusion, Changhai Hospital, Second Military Medical University, Shanghai 200433, P.R. China; 2 The University of Texas MD Anderson Cancer Center, Houston, TX 77030, USA; 3 Department of Internal Medicine, Emergency Center, East Hospital, Tongji University School of Medicine, Shanghai 200120, P.R. China

**Keywords:** interleukin-21, serum, interleukin-21 receptor, cluster of differentiation 8, diffuse large B-cell lymphoma

## Abstract

Interleukin 21 (IL-21) and its receptor, IL-21R, play a key role in innate and adaptive immunity. In the present study, the effect of IL-21 and IL-21R on the pathogenesis of diffuse large B-cell lymphoma (DLBCL) was investigated. The serum levels of IL-21 were detected by enzyme-linked immunosorbent assay, and the expression of IL-21R on CD8^+^ T cells was examined through flow cytometry. The data showed that the serum level of IL-21 was significantly decreased in the patients with DLBCL compared with the healthy controls (P<0.001), whereas the expression of IL-21R was clearly elevated on the CD8^+^ T cells in the patients with DLBCL. Further analyses revealed that the downregulation of the IL-21 serum level was correlated with an increased tumor stage of DLBCL, while the expression of IL-21R on the CD8^+^ T cells was positively correlated with the tumor stage. Also, the serum level of IL-21 and the proportion of IL-21R on the CD8^+^ T cells were negatively correlated in the patients. Notably, it was identified that the proportion of IL-21R on the CD8^+^ T cells, but not the serum level of IL-21, was significantly upregulated in the patients with bone-marrow involvement and B symptoms. These results indicate that IL-21 and IL-21R may be involved in the pathogenesis of DLBCL, in which IL-21R may reflect the progression of the disease more accurately than the serum level of IL-21.

## Introduction

Interleukin 21 (IL-21) belongs to the type I cytokine family and plays critical roles in the human immune system. IL-21 was originally believed to be produced by cluster of differentiation 4-positive (CD4^+^) T cells, among which, T-follicular helper cells could be a major source. Previous studies have shown that IL-21 is also produced by Th17 cells and by natural killer T cells, and that IL-21 mRNA can be detected in stromal cells in the lymph nodes. All these studies indicate the significance of IL-21 in innate and adaptive immune responses (1–3).

The IL-21 receptor (IL-21R) contains six tyrosines in the human cytoplasmic domains, Y281, Y361, Y369, Y397, Y317 and Y510 (4,5). Previous studies have reported that IL-21R and the common γ-chain (γc; CD132) function together as a heterodimer for IL-21 (6,7). Upon binding to the IL-21R expressed on cells lacking the γc, IL-21 is unable to transduce any intracytoplasmic signals, whereas in γc-transfected cells, IL-21 binds to the IL-21R and then activates signals downstream (7). Furthermore, a study on chemical cross-linking has previously demonstrated the direct binding of IL-21 to the γc (7). The expression of the IL-21R complex can be detected in various lymphoid organs, including the spleen and thymus. IL-21R is also expressed on T cells, B cells, dendritic cells, natural killer cells, keratinocytes and macrophages. A further study revealed that the functional IL-21R is expressed on CD4^+^ T cells and CD8^+^ T cells, and that this expression can be upregulated by the activation of the T cell receptor (3).

IL-21 plays significant roles in antitumor activity through the regulation of CD8^+^ T cells. It has been shown that IL-21 promotes CD8^+^ T-cell dependent tumor responses against solid tumors in a mouse model (8,9). IL-21 therapy in mice increases the number of tumor-infiltrating CD8^+^ T cells, expands the number of tumor-specific CD8^+^ T cells and protects the IL-21-treated mice against a recurrence of the same tumor (9). IL-21 treatment in humans causes the increased expression of perforin and granzyme B in CD8^+^ T cells, which induce apoptosis of B-cell chronic lymphocytic leukemia cells (10,11). These data indicate that CD8^+^ T cells may be incorporated into the IL-21-based therapies of hematological cancers. In the present study, the serum level of IL-21 and the expression of IL-21 on IL-21R with the pathogenesis of diffuse large B-cell lymphoma (DLBCL) was investigated.

## Materials and methods

### Study subjects

This study included 72 patients with a confirmed diagnosis of DLBCL and 62 healthy controls. The diagnosis of DLBCL was confirmed by histopathological examinations and, in the majority of cases, by supplementary immunohistochemistry analyses. The patient group was categorized according to the Ann Arbor staging system. The control group was recruited from healthy subjects who came to the same hospital for general health exams. All the control subjects were matched with the patient population based on age, gender and area of residence. Subjects who were relatives were excluded. All patients and controls were consecutively recruited from the East Hospital and Changhai Hospital (Shanghai, China), and were of Han Chinese ethnicity. Written informed consent was obtained from each subject. The study was approved by the Ethics Committee Board of Changhai Hospital (2005116) and Shanghai East Hospital (0582).

### Serum assay

Serum samples were collected from all 72 DLBCL patients and the 62 healthy controls. All samples were immediately stored at −80°C. Serum IL-21 was assessed using enzyme-linked immunosorbent assay (eBioscience, Inc., San Diego, CA, USA), with a detection threshold of 50 pg/ml.

### Flow cytometric analysis

The monoclonal antibodies (MoAbs) used in the present study included fluorescein isothiocyanate-conjugated anti-CD3 (BD Pharmingen; BD Biosciences, San Diego, CA, USA), phycoerythrin-conjugated anti-CD8 [clone RPA-T8, mouse immunoglobulin G (IgG1); BD Pharmingen], peridinin chlorophyll protein complex-conjugated goat anti-mouse IgG MoAb (BD Pharmingen) and anti-human IL-21R MoAb (mouse IgG1; R&D Systems, Minneapolis, MN, USA). Peripheral blood mononuclear cells were resuspended in phosphate-buffered saline supplemented with 0.2% bovine serum albumin and 0.1% NaN_3_ at a concentration of 1×10^6^ cells/ml. The cells were then stained with optimal concentrations of fluorochrome-labeled MoAbs and isotype-matched control MoAb, fixed in 3% paraformaldehyde buffer and determined by flow cytometry (FACSCalibur) using CellQuest software (BD Biosciences).

### Statistical analysis

The SPSS statistical software package version 13.0 (SPSS, Inc., Chicago, IL, USA) was used for the statistical analysis. Student’s t-test and the Mann-Whitney non-parametric U test were used for comparison. Pearson’s correlation analysis was used to calculate the correlation coefficient. P<0.05 was considered to indicate a statistically significant difference.

## Results

### Clinical characteristics of the study subjects

Selected characteristics of the 72 patients with DLBCL and 62 controls are presented in Table I. The cases and controls did not reveal any statistical significance with regard to age (P>0.05) and gender (P>0.05). Of the 72 patients, eight were in Ann Arbor stage I, 10 were in stage II, 25 were in stage III and 29 were in stage IV. A total of 18 cases exhibited bone marrow involvement and 30 patients presented with B symptoms.

### Serum level of IL-21 and expression of IL-21R on CD8^+^ T cells in cases and controls

**Figure 1:**
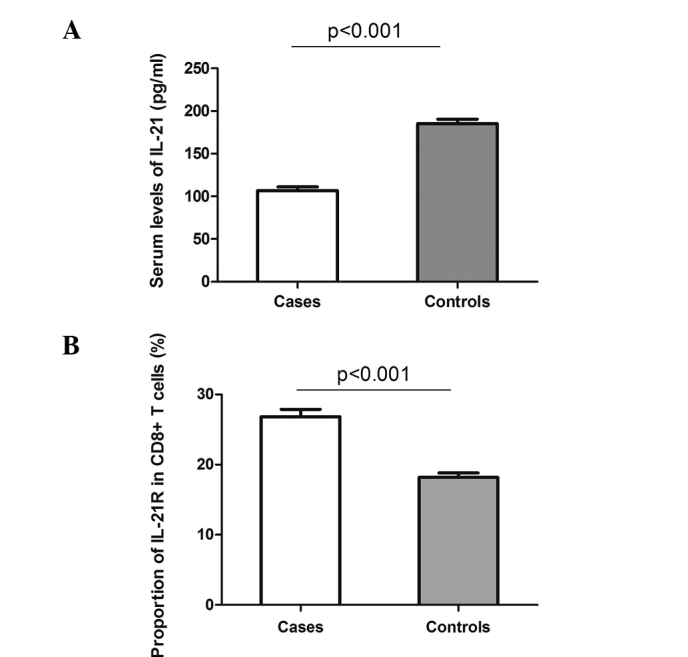
Serum levels of IL-21 and IL-21R expression on peripheral CD8^+^ T cells were significantly altered in the patients with DLBCL compared with the healthy controls. (A) The serum levels of IL-21 were decreased in the patients compared with the controls. (B) The percentage of IL-21R on the CD8^+^ T cells was increased in the patients compared with the controls. Data were calculated from 72 patients and 62 healthy controls. P-values are shown. IL-21, interleukin 21; IL-21R, IL-21 receptor; CD8, cluster of differentiation 8; DLBCL, diffuse large B-cell lymphoma.

The serum levels of IL-21 were investigated in the 72 cases and 62 controls. As shown in Fig. 1A, a decreased level of IL-21 was detected in the patients with DLBCL compared with the controls (mean ± standard error of the mean, 106.8±4.4 vs. 185.3±5.3 pg/ml; P<0.001). However, when comparing the expression of IL-21R on the CD8^+^ T cells between the cases and controls, a clearly elevated proportion of IL-21R was observed on the CD8^+^ T cells in the DLBCL cases (26.8±1.1%) compared with the controls (18.2±0.6%; Fig. 1B). These data indicate a potential involvement of IL-21 and IL-21R in the pathogenesis of DLBCL.

### Serum level of IL-21 and expression of IL-21R on CD8^+^ T cells in patients with various tumor stages

**Figure 2:**
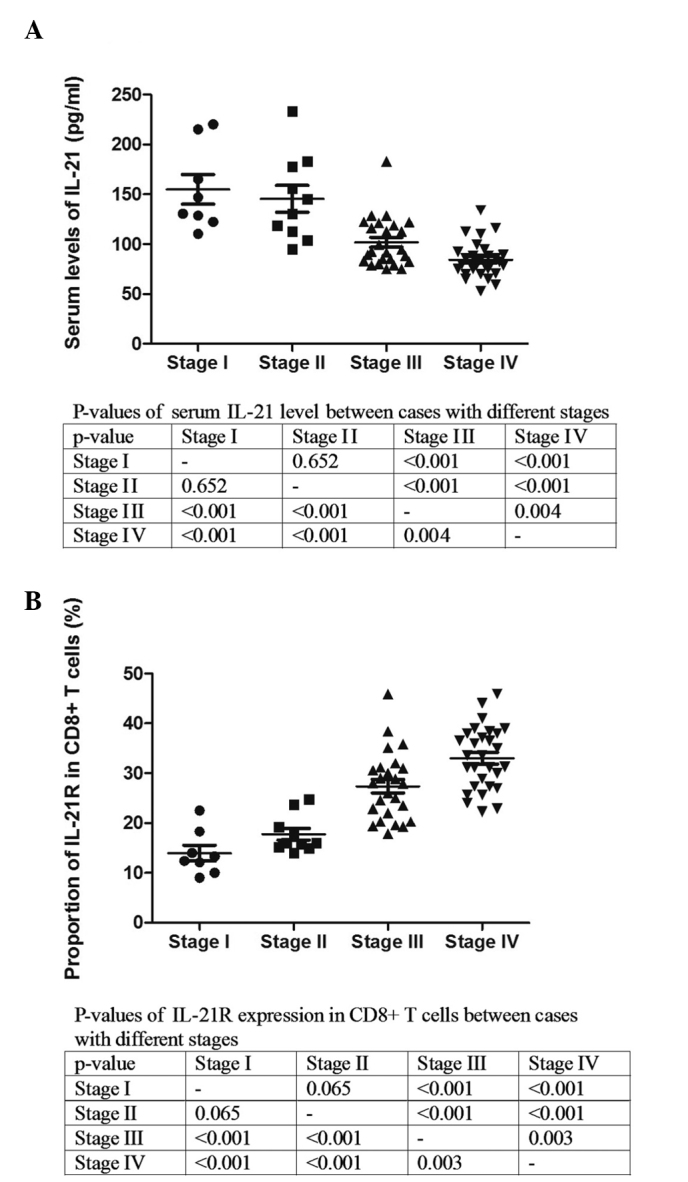
(A) Serum levels of IL-21 and (B) IL-21R expression on peripheral CD8^+^ T cells in the patients with DLBCL with different Ann Arbor stages. Each symbol represents a subject. P-values of each comparison are shown in the tables below. IL-21, interleukin 21; IL-21R, IL-21 receptor; CD8, cluster of differentiation 8; DLBCL, diffuse large B-cell lymphoma.

The Ann Arbor staging system is a standardized way for a cancer care team to summarize information with regard to how far a cancer has spread (11). Stage I is indicative of a cancer that is located in a single region, typically one lymph node and the surrounding area, whereas stage IV is indicative of the diffuse or disseminated involvement of one or more extralymphatic organs. The serum level of IL-21 and the expression of IL-21R on the CD8^+^ T cells were analyzed in the patients with various stages (Fig. 2). The serum level of IL-21 was significantly lower in the patients with stage III and IV compared with those with stages I and II (Fig. 2A). The analysis of IL-21R demonstrated that the expression of IL-21R on the CD8^+^ T cells was clearly elevated with increasing stage (Fig. 2B). These results indicate that IL-21 and IL-21R could be associated with the progression of DLBCL.

### Correlation between serum level of IL-21 and expression of IL-21R on CD8^+^ T cells in DLBCL

**Figure 3:**
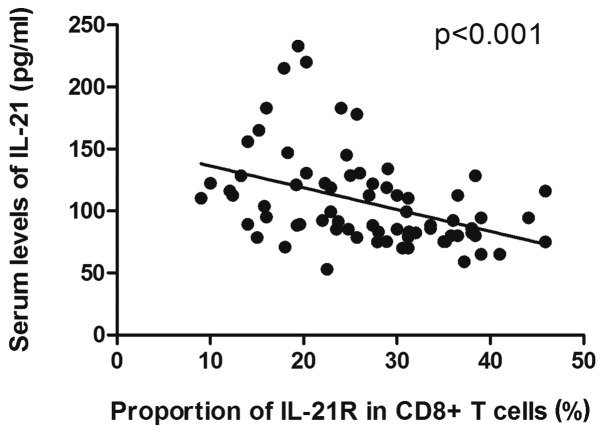
Analysis of the correlation between serum levels of IL-21 and IL-21R expression on peripheral CD8^+^ T cells in the patients with DLBCL. Each symbol represents a subject. The P-value is shown. IL-21, interleukin 21; IL-21R, IL-21 receptor; CD8, cluster of differentiation 8; DLBCL, diffuse large B-cell lymphoma.

Since a decreased level of serum IL-21, but increased expression of IL-21R on CD8^+^ T cells, was observed in the DLBCL samples, and due to the diverse correlations with disease progression, we hypothesized that a potential correlation existed between the serum level of IL-21 and the expression of IL-21R on the CD8^+^ T cells in the patients with DLBCL. The results data showed that the decrease in the serum level of IL-21 was significantly correlated with the increase in the expression of IL-21R on the CD8^+^ T cells in the patients with DLBCL (P<0.001; Fig. 3).

### Serum level of IL-21 and expression of IL-21R on CD8^+^ T cells in patients with systemic symptoms

**Figure 4:**
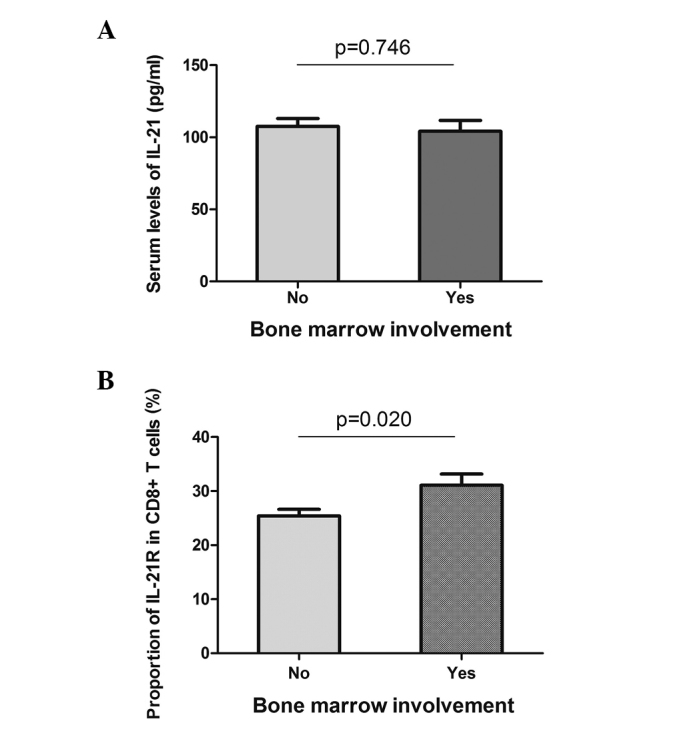
(A) Serum levels of IL-21 and (B) IL-21R expression on peripheral CD8^+^ T cells in the patients with DLBCL with a different status of bone marrow involvement. P-values of each comparison are shown. IL-21, interleukin 21; IL-21R, IL-21 receptor; CD8, cluster of differentiation 8; DLBCL, diffuse large B-cell lymphoma.

**Figure 5:**
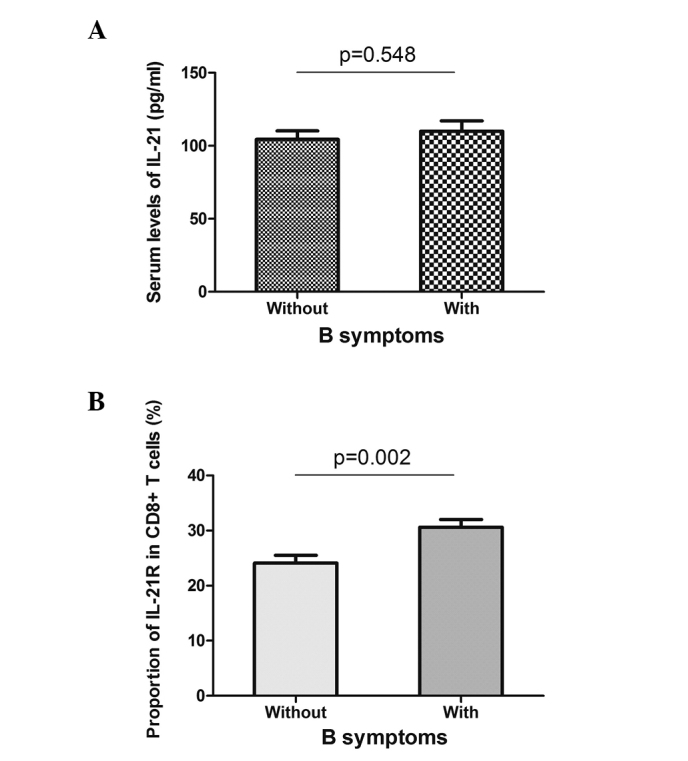
(A) Serum levels of IL-21 and (B) IL-21R expression on peripheral CD8^+^ T cells in the patients with DLBCL with a different status of B symptoms. P-values of each comparison are shown. IL-21, interleukin 21; IL-21R, IL-21 receptor; CD8, cluster of differentiation 8; DLBCL, diffuse large B-cell lymphoma.

Bone marrow involvement and B symptoms are two systemic symptoms of DLBCL, and they indicate a poor prognosis of the disease. The serum level of IL-21 and the expression of IL-21R on the CD8^+^ T cells were analyzed in the patients with or without systemic symptoms. With regard to bone marrow involvement, the serum level of IL-21 did not reveal a difference between the two groups (Fig. 4A), while the expression of IL-21R on the CD8^+^ T cells was higher in the positive group (31.1±2.1 vs. 25.4±1.2%, P=0.020; Fig. 4B). Similarly, the patients with or without B symptoms did not present different serum levels of IL-21, but showed a higher expression of IL-21R when B symptoms were present (Fig. 5). These data indicated that the expression of IL-21R on CD8^+^ T cells may be used as a prognostic marker for DLBCL.

## Discussion

In the present study, IL-21 and its receptor were analyzed in CD8^+^ T cells, and it was identified that IL-21/IL-21R are closely involved in the development and progression of DLBCL.

Several studies conducted on murine models have confirmed the antitumor role of IL-21 (12–14). DLBCL is a heterogeneous disease with multiple subtypes and varying clinical outcomes. It has been reported that IL-21R expression is positive on primary CD10^+^ DLBCL cells and DLBCL cell lines (15–17). IL-21 is known to induce apoptosis in *de novo* DLBCL primary tumors, however, it may not effect the viability of human healthy B cells (15). Additionally, IL-21 is able to induce tumor regression and increase the survival of mice with xenograft DLBCL tumors (17). IL-21 stimulates the apoptosis of the DLBCL cell line, CRL-2632, by activating JAK1, JAK3, STAT1 and STAT3 (15). In addition, it appears that the anti-lymphoma effects of IL-21 are dependent on a mechanism involving the IL-21-activated STAT3 upregulation of c-Myc, in which c-Myc is a highly prognostic marker in DLBCL (17,18). The present study confirmed the critical role of IL-21 in the development of DLBCL by identifying a decreased serum level of IL-21 in the patient group (Fig. 1A). A negative correlation was also found between the serum level of IL-21 and the tumor stage, indicating the involvement of this cytokine in the progression of the disease (Fig. 2A).

The effects of IL-21 on CD8^+^ T cells are varied. IL-21 increases the proliferation of murine or human mature T cells stimulated with anti-CD3 or its antigen (19). In conjunction with T-cell receptor stimulation and other common γc cytokines, including IL-7 and IL-15, IL-21 is able to augment the proliferation and differentiation of human and mouse CD8^+^ T cells into potent cytolytic effectors (19,20). In the present study, an elevated level of IL-21R was observed on the peripheral CD8^+^ T cells in the DLBCL cases (Fig. 2B), and this positively correlated with the tumor grade. Notably, the level of IL-21R on the peripheral CD8^+^ T cells, but not the serum level of IL-21, was associated with bone marrow involvement and B symptoms, indicating that IL-21R on peripheral CD8^+^ T cells may play more significant roles in the progression of DLBCL.

In conclusion, the present study identified a decreased level of serum IL-21, but an increased level of IL-21R expression on the CD8^+^ T cells in the patients with DLBCL. Additionally, the analyses revealed opposite correlations between IL-21 and IL-21R with disease progression, in which IL-21R on CD8^+^ T cells may further reflect the prognosis of the disease. These data shed light on understanding the pathogenesis of DLBCL and provide knowledge for the use of IL-21 as a novel therapy.

## Figures and Tables

**Table I:** Characteristics of the patients (n=72) and control subjects (n=62).

Characteristics	Patients, n (%)	Controls, n (%)	P-value
Age, years			
≥60	45 (62.5)	39 (62.9)	>0.05
<60	27 (37.5)	23 (37.1)	
Gender			
Male	42 (58.3)	34 (54.8)	>0.05
Female	30 (41.7)	28 (45.2)	
Ann Arbor stage			
I	8 (11.1)		
II	10 (13.9)		
III	25 (34.7)		
IV	29 (40.3)		
Bone marrow involvement			
No	54 (75.0)		
Yes	18 (25.0)		
B symptoms			
With	30 (41.7)		
Without	42 (58.3)		
